# Supplementary figures and images for: Expression and prognostic analyses of ITGA11, ITGB4 and ITGB8 in human non-small cell lung cancer
Source: PeerJ. 2019 Dec 20;7:e8299. doi: 10.7717/peerj.8299 (PMC6927340; doi:10.7717/peerj.8299)

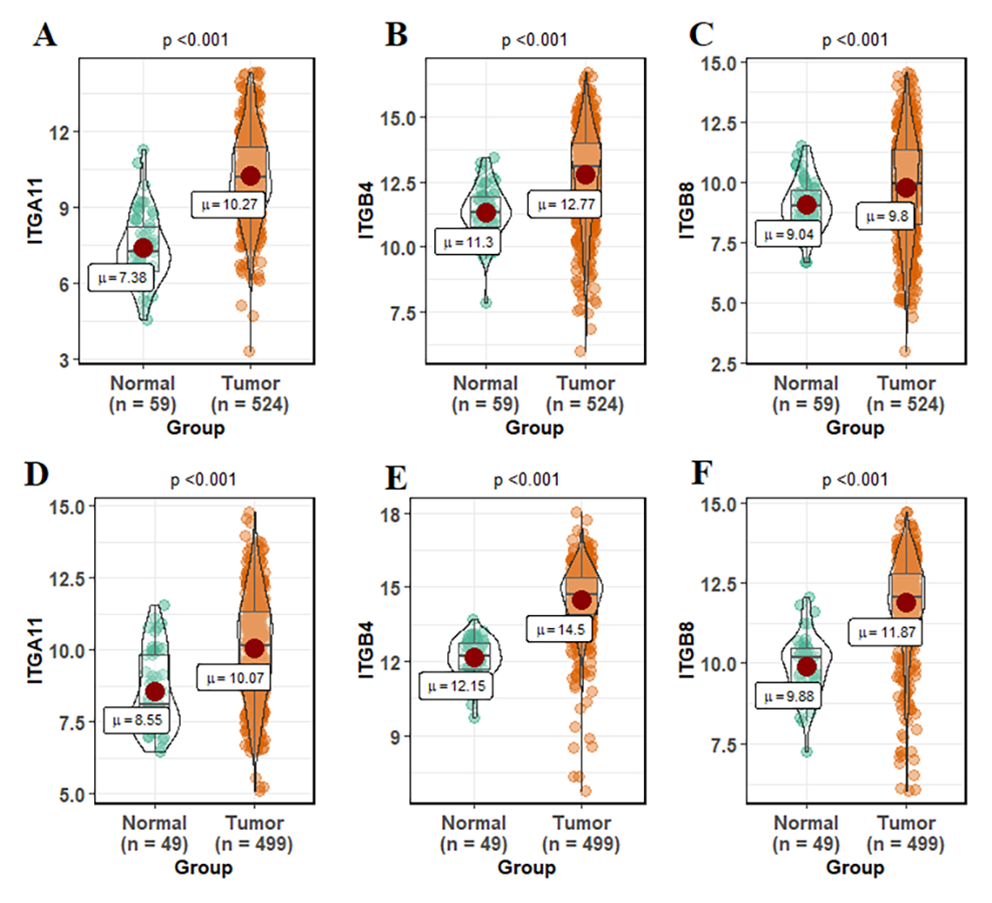

Supplement: Figure S1 [file peerj-07-8299-s001.png]

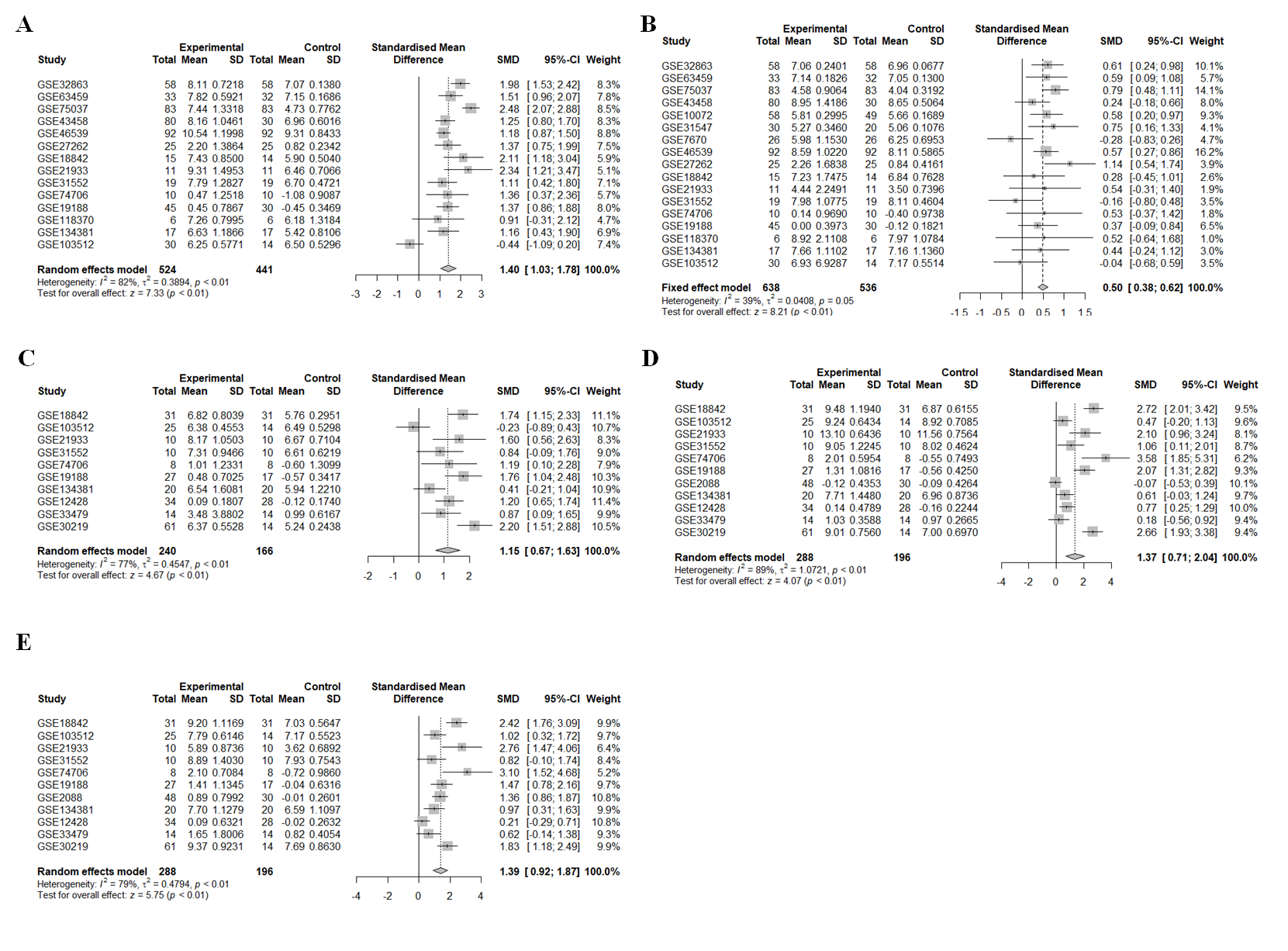

Supplement: Figure S2 — (A–B) The forest plots of overall analysis of ITGA11 and ITGB8 between LUAD patients and normal controls, respectively. (C–E) The forest plots of overall analysis of ITGA11, ITGB4 and ITGB8 between LUSC patients and normal controls, respectively. [file peerj-07-8299-s002.png]

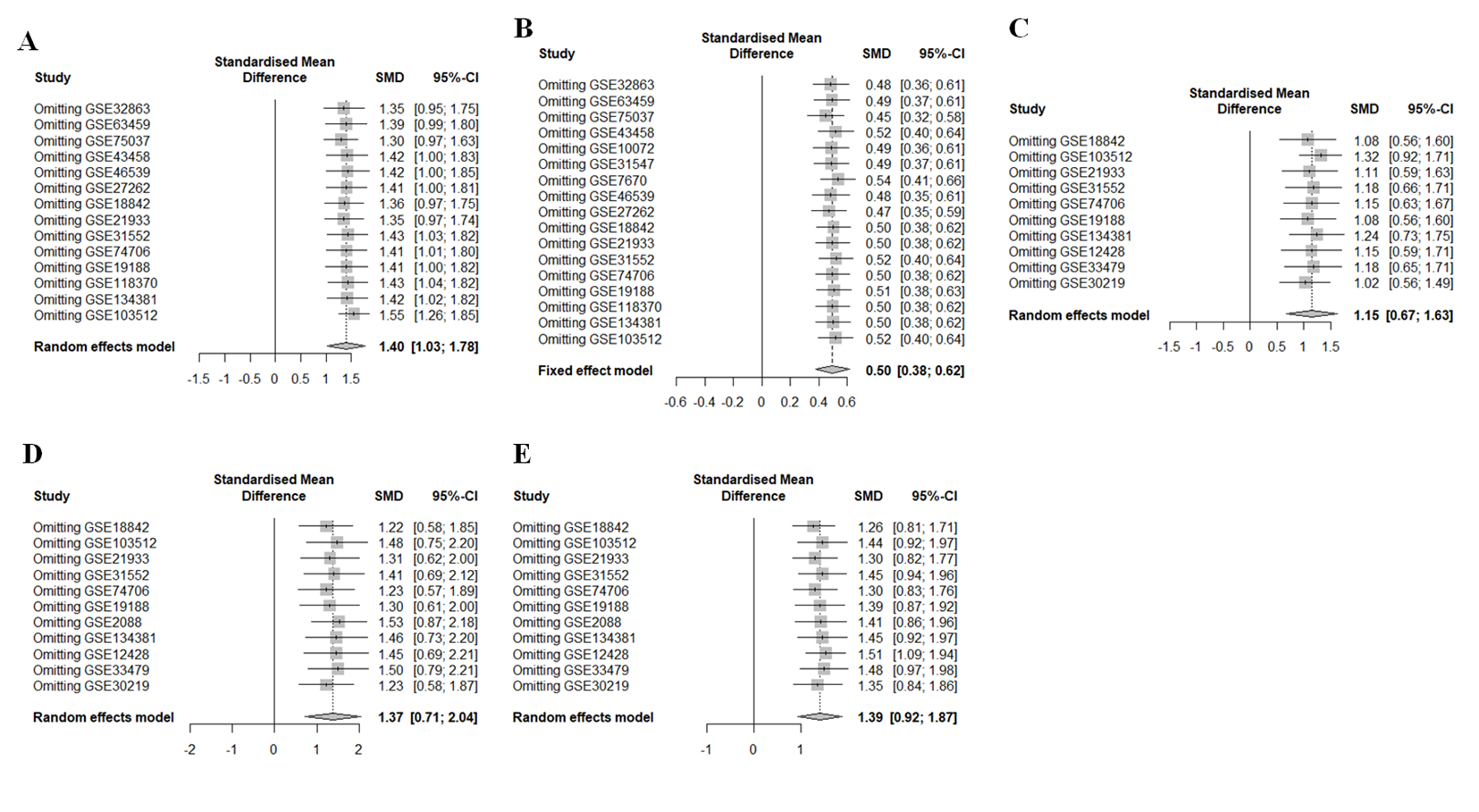

Supplement: Figure S3 — (A–B) Sensitivity analysis for the enrolled GEO datasets in analyzing ITGA11 and ITGB8 expression between LUAD patients and normal controls, respectively. (C–E) Sensitivity analysis for the enrolled GEO datasets in analyzing ITGA11, ITGB4 and ITGB8 expression between LUSC patients and normal controls, respectively. [file peerj-07-8299-s003.png]

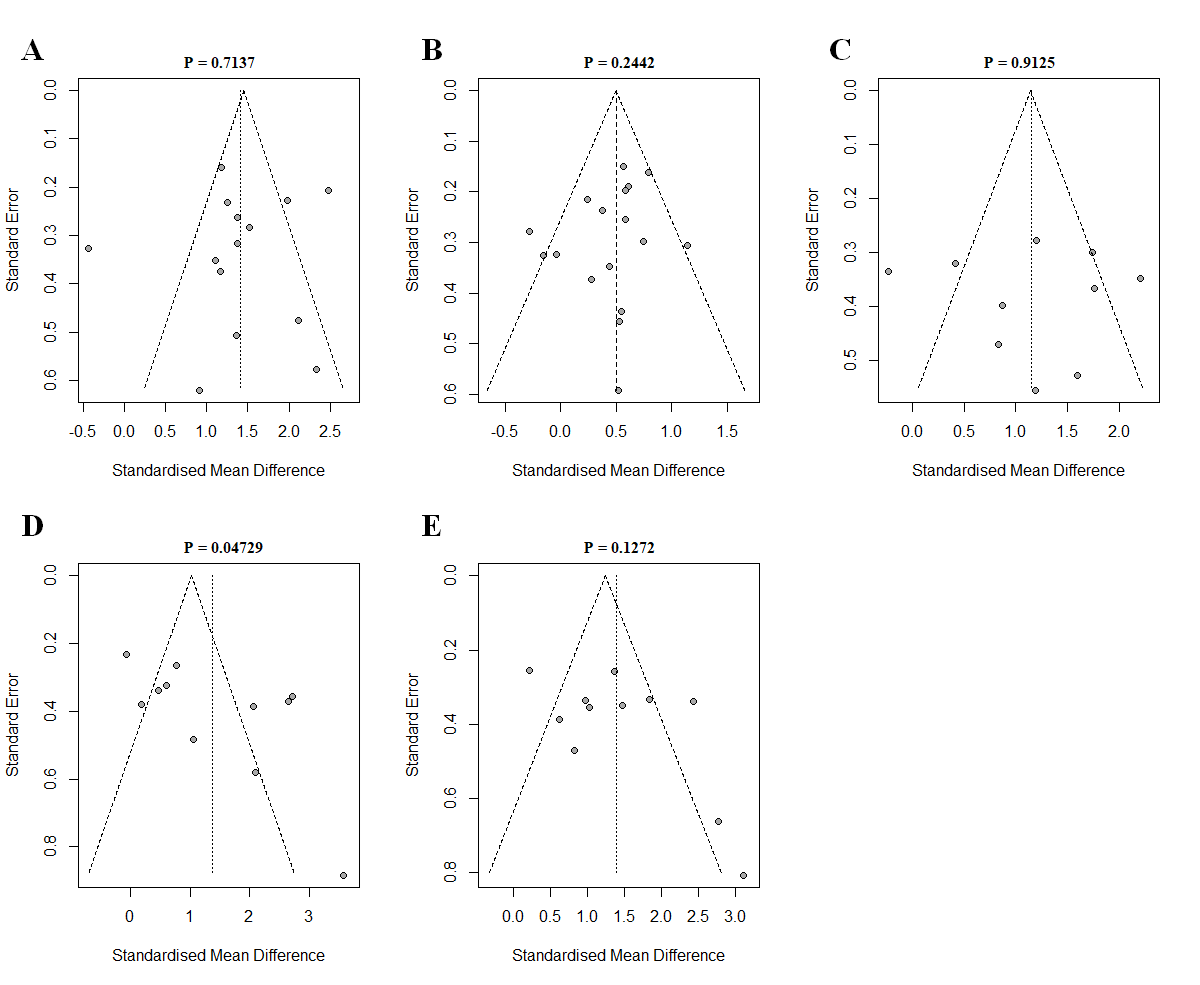

Supplement: Figure S4 — (A–B) Funnel plots and Egger’s test for the enrolled GEO datasets in analyzing ITGA11 and ITGB8 expression between LUAD tissues and normal controls, respectively. (C–E) Funnel plots and Egger’s test for the enrolled GEO datasets in analyzing ITGA11, ITGB4 and ITGB8 expression between LUSC tissues and normal controls, respectively. [file peerj-07-8299-s004.png]

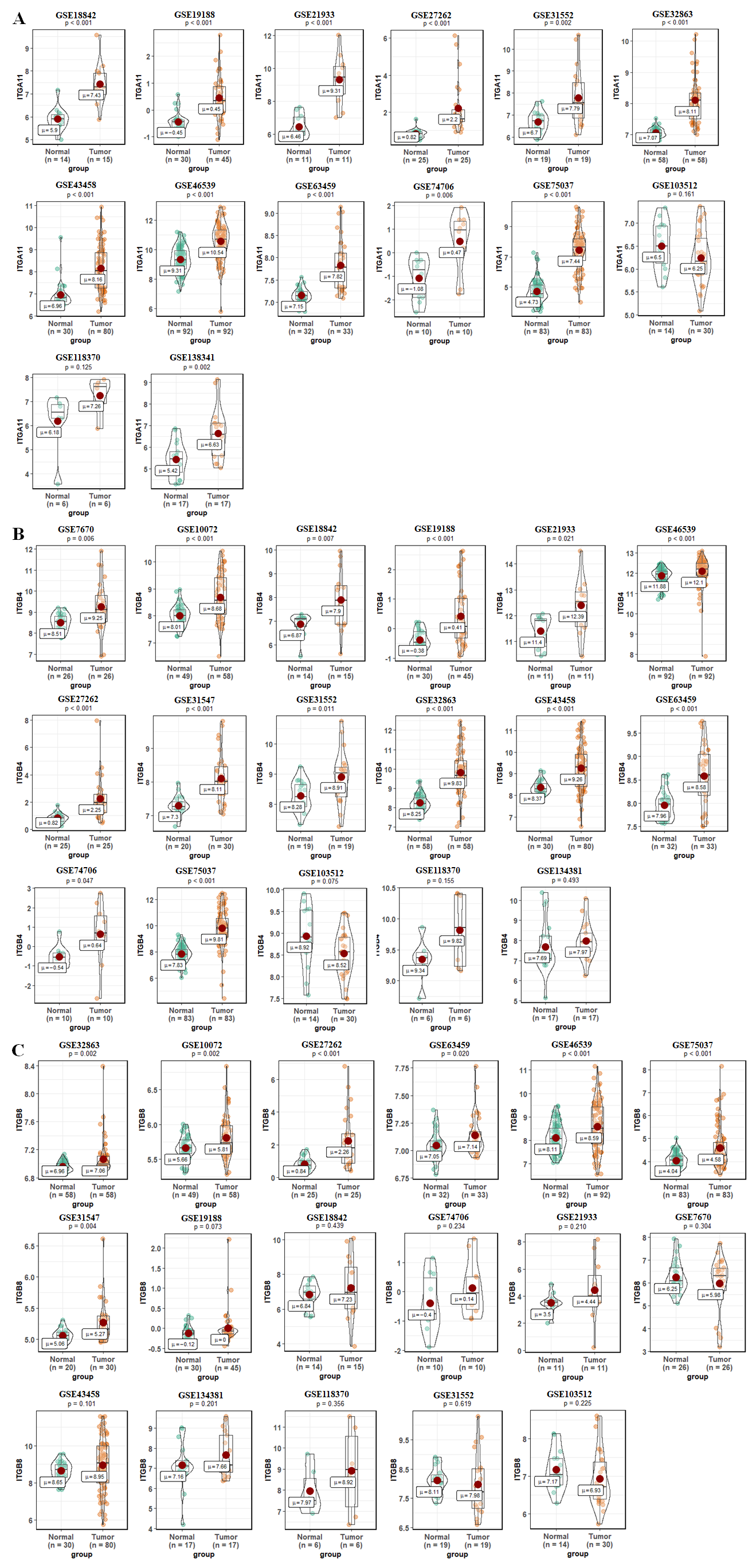

Supplement: Figure S5 — (A–C) The expression levels of ITGA11, ITGB4 and ITGB8 between LUAD and normal tissues for each GEO dataset, respectively. [file peerj-07-8299-s005.png]

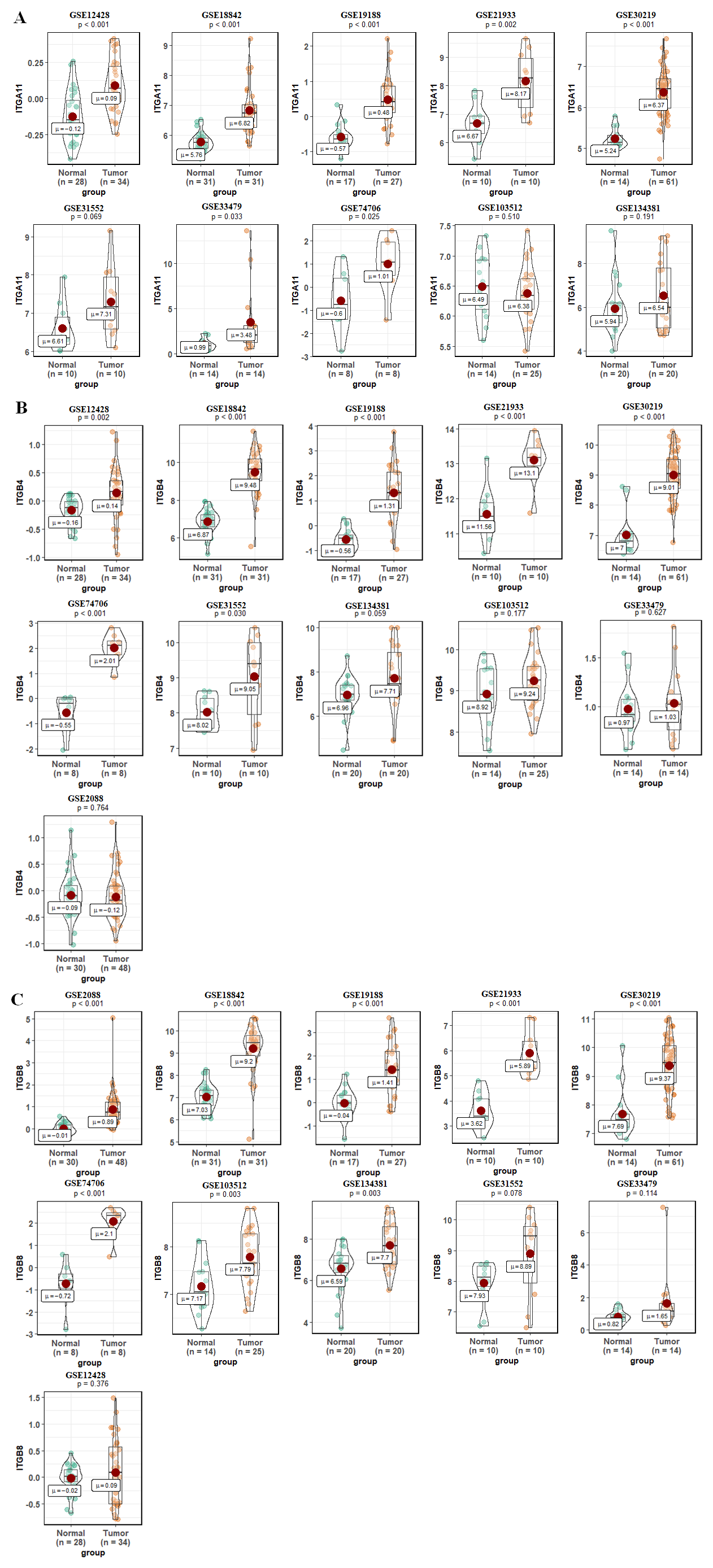

Supplement: Figure S6 — (A–C) The expression levels of ITGA11, ITGB4 and ITGB8 between LUSC and normal tissues for each GEO dataset, respectively. [file peerj-07-8299-s006.png]

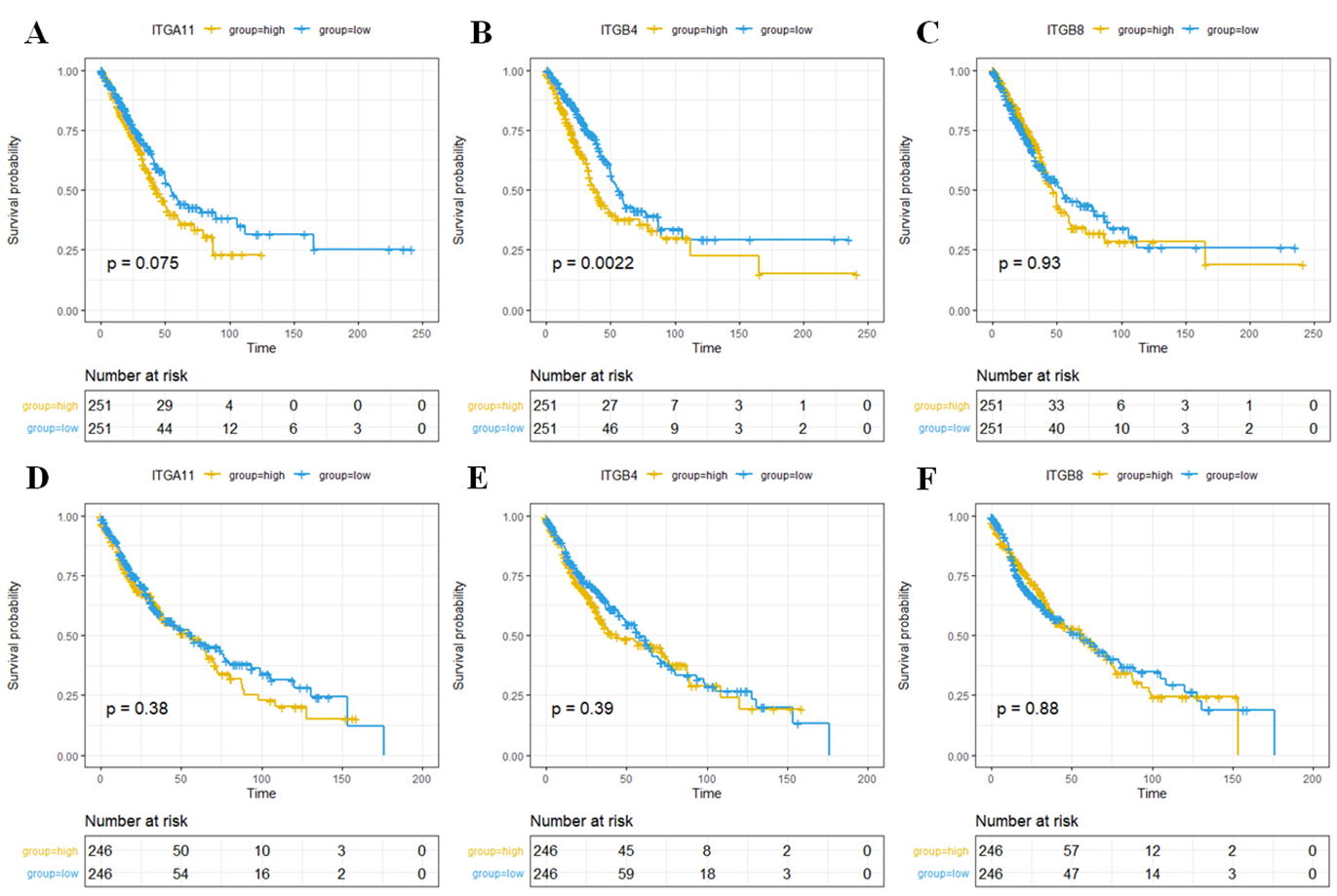

Supplement: Figure S7 — Survival curves of OS based on the high and low expression of ITGA11, ITGB4 and ITGB8 in LUAD (A–C) and LUSC (D–F), respectively. [file peerj-07-8299-s007.png]

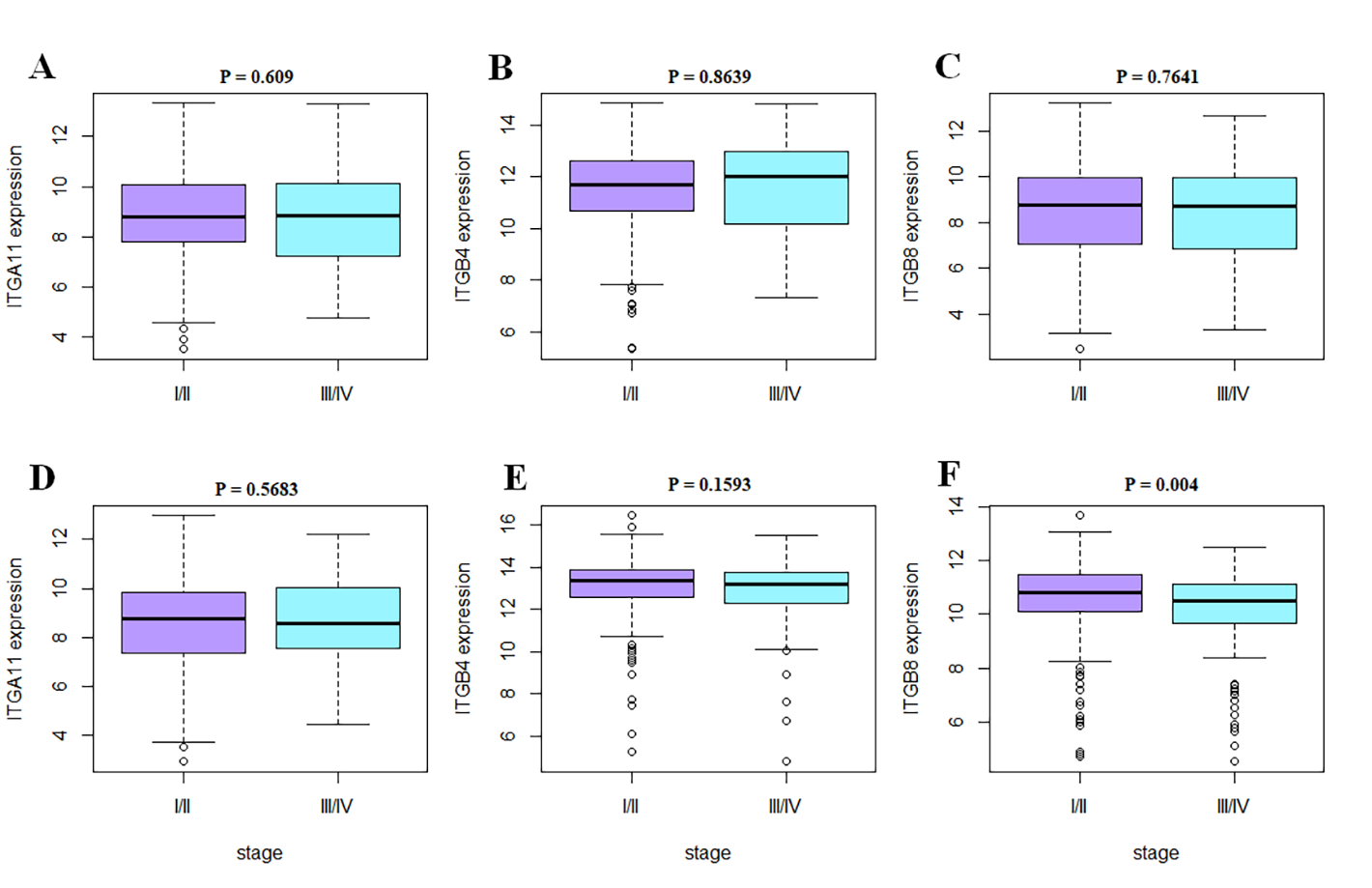

Supplement: Figure S8 — The association between the expression levels of ITGA11, ITGB4 and ITGB8 and tumor stages in LUAD (A–C) and LUSC (D–F), respectively. [file peerj-07-8299-s008.png]

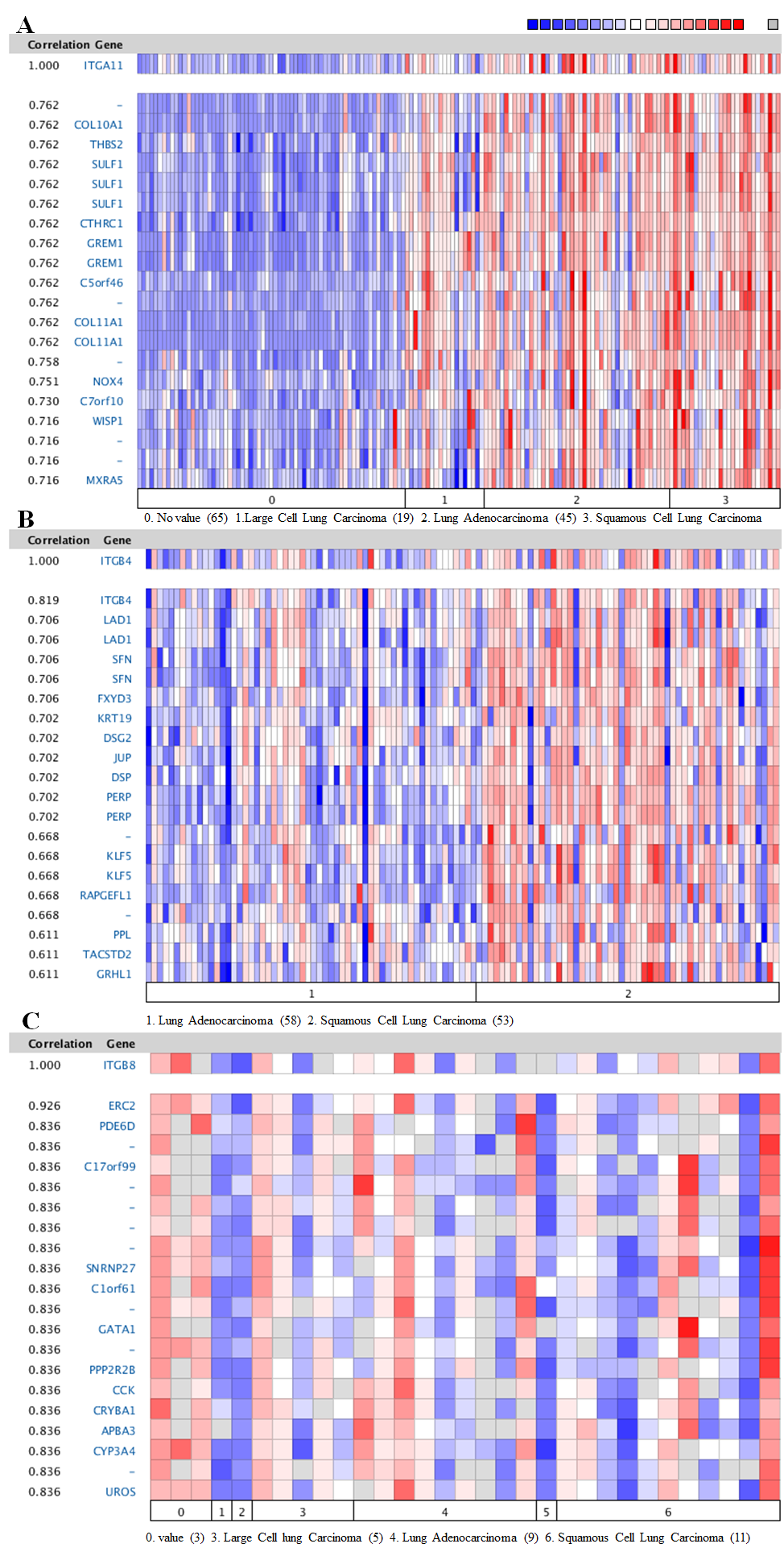

Supplement: Figure S9 [file peerj-07-8299-s009.png]

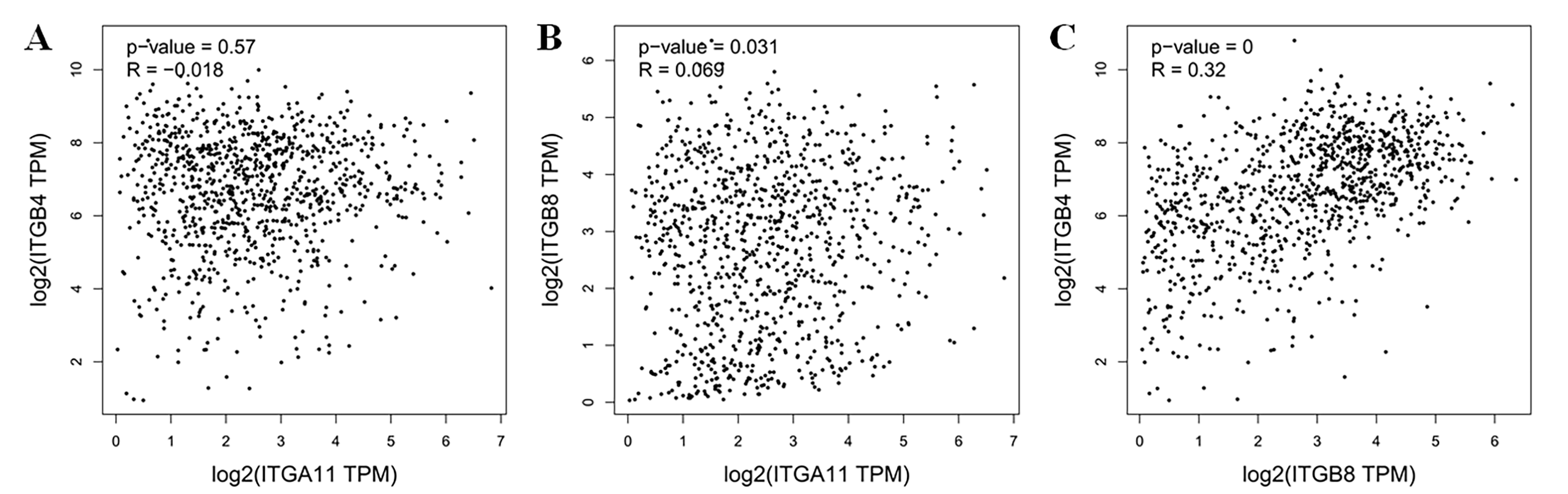

Supplement: Figure S10 [file peerj-07-8299-s010.png]
