# Supplementary material for: Expression and prognostic analyses of ITGA11, ITGB4 and ITGB8 in human non-small cell lung cancer
Source: PeerJ. 2019 Dec 20;7:e8299. doi: 10.7717/peerj.8299 (PMC6927340; doi:10.7717/peerj.8299)
Supplement: Table S1 [file peerj-07-8299-s011.docx]

| GEO dataset | Year | Platform | LUAD | Controls | LUSC | Controls | Sample type in patients | Sample type in controls | Reference |
| --- | --- | --- | --- | --- | --- | --- | --- | --- | --- |
| GSE32863 | 2012 | GPL6884 | 58 | 58 |  |  | Cancer tissue | Adjacent non-tumor tissues | (Selamat et al. 2012) |
| GSE63459 | 2015 | GPL6883 | 33 | 32 |  |  | Cancer tissue | Adjacent non-tumor tissues | (Robles et al. 2015) |
| GSE75037 | 2016 | GPL6884 | 83 | 83 |  |  | Cancer tissue | Adjacent non-tumor tissues | (Girard et al. 2016) |
| GSE43458 | 2013 | GPL6244 | 80 | 30 |  |  | Cancer tissue | Adjacent non-tumor tissues | (Kabbout et al. 2013) |
| GSE10072 | 2008 | GPL96 | 58 | 49 |  |  | Cancer tissue | Healthy lung tissues | (Landi et al. 2008) |
| GSE31547 | 2011 | GPL96 | 30 | 20 |  |  | Cancer tissue | Adjacent non-tumor tissues | Not published |
| GSE7670 | 2007 | GPL96 | 26 | 26 |  |  | Cancer tissue | Adjacent non-tumor tissues | (Chen et al. 2009; Su et al. 2007) |
| GSE46539 | 2015 | GPL6883 | 92 | 92 |  |  | Cancer tissue | Adjacent non-tumor tissues | (Chang et al. 2017; Chen et al. 2015) |
| GSE27262 | 2013 | GPL570 | 25 | 25 |  |  | Cancer tissue | Adjacent non-tumor tissues | (Wei et al. 2014; Wei et al. 2012) |
| GSE18842 | 2010 | GPL570 | 15 | 14 | 31 | 31 | Cancer tissue | Adjacent non-tumor tissues | (Sanchez-Palencia et al. 2011) |
| GSE21933 | 2013 | GPL6254 | 11 | 11 | 10 | 10 | Cancer tissue | Adjacent non-tumor tissues | (Lo et al. 2012) |
| GSE31552 | 2011 | GPL6244 | 19 | 19 | 10 | 10 | Cancer tissue | Adjacent non-tumor tissues | (Lin et al. 2014) |
| GSE74706 | 2016 | GPL13497 | 10 | 10 | 8 | 8 | Cancer tissue | Adjacent non-tumor tissues | (Marwitz et al. 2016) |
| GSE19188 | 2010 | GPL570 | 45 | 30 | 27 | 17 | Cancer tissue | Adjacent non-tumor tissues | (Hou et al. 2010) |
| GSE118370 | 2019 | GPL570 | 6 | 6 |  |  | Cancer tissue | Adjacent non-tumor tissues | (Xu et al. 2018) |
| GSE134381 | 2019 | GPL11532 | 17 | 17 | 20 | 20 | Cancer tissue | Adjacent non-tumor tissues | (Hoang et al. 2019) |
| GSE103512 | 2017 | GPL13158 | 30 | 14 | 25 | 14 | Cancer tissue | Adjacent non-tumor tissues | (Brouwer-Visser et al. 2018) |
| GSE2088 | 2009 | GPL962 |  |  | 48 | 30 | Cancer tissue | Healthy lung tissues | (Fujiwara et al. 2012) |
| GSE12428 | 2008 | GPL1708 |  |  | 34 | 28 | Cancer tissue | Healthy lung tissues | (Boelens et al. 2009) |
| GSE33479 | 2014 | GPL6480 |  |  | 14 | 14 | Cancer tissue | Healthy lung tissues | (Mascaux et al. 2019) |
| GSE30219 | 2013 | GPL570 |  |  | 61 | 14 | Cancer tissue | Healthy lung tissues | (Rousseaux et al. 2013) |

**References:**

Chang IS, Jiang SS, Yang JC, Su WC, Chien LH, Hsiao CF, Lee JH, Chen CY, Chen CH, Chang GC, Wang Z, Lo FY, Chen KY, Wang WC, Chen YM, Huang MS, Tsai YH, Su YC, Hsieh WS, Shih WC, Shieh SH, Yang TY, Lan Q, Rothman N, Chen CJ, Chanock SJ, Yang PC, and Hsiung CA. 2017. Genetic Modifiers of Progression-Free Survival in Never-Smoking Lung Adenocarcinoma Patients Treated with First-Line Tyrosine Kinase Inhibitors. *Am J Respir Crit Care Med* 195:663-673. 10.1164/rccm.201602-0300OC

Chen CH, Lai JM, Chou TY, Chen CY, Su LJ, Lee YC, Cheng TS, Hong YR, Chou CK, Whang-Peng J, Wu YC, and Huang CY. 2009. VEGFA upregulates FLJ10540 and modulates migration and invasion of lung cancer via PI3K/AKT pathway. *PLoS One* 4:e5052. 10.1371/journal.pone.0005052

Chen KY, Hsiao CF, Chang GC, Tsai YH, Su WC, Chen YM, Huang MS, Tsai FY, Jiang SS, Chang IS, Chen CY, Hsiung CA, Chen CJ, Yang PC, and Group GS. 2015. Estrogen Receptor Gene Polymorphisms and Lung Adenocarcinoma Risk in Never-Smoking Women. *J Thorac Oncol* 10:1413-1420. 10.1097/JTO.0000000000000646

Girard L, Rodriguez-Canales J, Behrens C, Thompson DM, Botros IW, Tang H, Xie Y, Rekhtman N, Travis WD, Wistuba, II, Minna JD, and Gazdar AF. 2016. An Expression Signature as an Aid to the Histologic Classification of Non-Small Cell Lung Cancer. *Clin Cancer Res* 22:4880-4889. 10.1158/1078-0432.CCR-15-2900

Kabbout M, Garcia MM, Fujimoto J, Liu DD, Woods D, Chow CW, Mendoza G, Momin AA, James BP, Solis L, Behrens C, Lee JJ, Wistuba, II, and Kadara H. 2013. ETS2 mediated tumor suppressive function and MET oncogene inhibition in human non-small cell lung cancer. *Clin Cancer Res* 19:3383-3395. 10.1158/1078-0432.CCR-13-0341

Landi MT, Dracheva T, Rotunno M, Figueroa JD, Liu H, Dasgupta A, Mann FE, Fukuoka J, Hames M, Bergen AW, Murphy SE, Yang P, Pesatori AC, Consonni D, Bertazzi PA, Wacholder S, Shih JH, Caporaso NE, and Jen J. 2008. Gene expression signature of cigarette smoking and its role in lung adenocarcinoma development and survival. *PLoS One* 3:e1651. 10.1371/journal.pone.0001651

Robles AI, Arai E, Mathe EA, Okayama H, Schetter AJ, Brown D, Petersen D, Bowman ED, Noro R, Welsh JA, Edelman DC, Stevenson HS, Wang Y, Tsuchiya N, Kohno T, Skaug V, Mollerup S, Haugen A, Meltzer PS, Yokota J, Kanai Y, and Harris CC. 2015. An Integrated Prognostic Classifier for Stage I Lung Adenocarcinoma Based on mRNA, microRNA, and DNA Methylation Biomarkers. *J Thorac Oncol* 10:1037-1048. 10.1097/JTO.0000000000000560

Selamat SA, Chung BS, Girard L, Zhang W, Zhang Y, Campan M, Siegmund KD, Koss MN, Hagen JA, Lam WL, Lam S, Gazdar AF, and Laird-Offringa IA. 2012. Genome-scale analysis of DNA methylation in lung adenocarcinoma and integration with mRNA expression. *Genome Res* 22:1197-1211. 10.1101/gr.132662.111

Su LJ, Chang CW, Wu YC, Chen KC, Lin CJ, Liang SC, Lin CH, Whang-Peng J, Hsu SL, Chen CH, and Huang CY. 2007. Selection of DDX5 as a novel internal control for Q-RT-PCR from microarray data using a block bootstrap re-sampling scheme. *BMC Genomics* 8:140. 10.1186/1471-2164-8-140

Boelens MC, van den Berg A, Fehrmann RS, Geerlings M, de Jong WK, te Meerman GJ, Sietsma H, Timens W, Postma DS, and Groen HJ. 2009. Current smoking-specific gene expression signature in normal bronchial epithelium is enhanced in squamous cell lung cancer. *J Pathol* 218:182-191. 10.1002/path.2520

Brouwer-Visser J, Cheng WY, Bauer-Mehren A, Maisel D, Lechner K, Andersson E, Dudley JT, and Milletti F. 2018. Regulatory T-cell Genes Drive Altered Immune Microenvironment in Adult Solid Cancers and Allow for Immune Contextual Patient Subtyping. *Cancer Epidemiol Biomarkers Prev* 27:103-112. 10.1158/1055-9965.EPI-17-0461

Fujiwara T, Hiramatsu M, Isagawa T, Ninomiya H, Inamura K, Ishikawa S, Ushijima M, Matsuura M, Jones MH, Shimane M, Nomura H, Ishikawa Y, and Aburatani H. 2012. ASCL1-coexpression profiling but not single gene expression profiling defines lung adenocarcinomas of neuroendocrine nature with poor prognosis. *Lung Cancer* 75:119-125. 10.1016/j.lungcan.2011.05.028

Hoang LT, Domingo-Sabugo C, Starren ES, Willis-Owen SAG, Morris-Rosendahl DJ, Nicholson AG, Cookson W, and Moffatt MF. 2019. Metabolomic, transcriptomic and genetic integrative analysis reveals important roles of adenosine diphosphate in haemostasis and platelet activation in non-small-cell lung cancer. *Mol Oncol*. 10.1002/1878-0261.12568

Hou J, Aerts J, den Hamer B, van Ijcken W, den Bakker M, Riegman P, van der Leest C, van der Spek P, Foekens JA, Hoogsteden HC, Grosveld F, and Philipsen S. 2010. Gene expression-based classification of non-small cell lung carcinomas and survival prediction. *PLoS One* 5:e10312. 10.1371/journal.pone.0010312

Lin J, Marquardt G, Mullapudi N, Wang T, Han W, Shi M, Keller S, Zhu C, Locker J, and Spivack SD. 2014. Lung cancer transcriptomes refined with laser capture microdissection. *Am J Pathol* 184:2868-2884. 10.1016/j.ajpath.2014.06.028

Lo FY, Chang JW, Chang IS, Chen YJ, Hsu HS, Huang SF, Tsai FY, Jiang SS, Kanteti R, Nandi S, Salgia R, and Wang YC. 2012. The database of chromosome imbalance regions and genes resided in lung cancer from Asian and Caucasian identified by array-comparative genomic hybridization. *BMC Cancer* 12:235. 10.1186/1471-2407-12-235

Marwitz S, Depner S, Dvornikov D, Merkle R, Szczygiel M, Muller-Decker K, Lucarelli P, Wasch M, Mairbaurl H, Rabe KF, Kugler C, Vollmer E, Reck M, Scheufele S, Kroger M, Ammerpohl O, Siebert R, Goldmann T, and Klingmuller U. 2016. Downregulation of the TGFbeta Pseudoreceptor BAMBI in Non-Small Cell Lung Cancer Enhances TGFbeta Signaling and Invasion. *Cancer Res* 76:3785-3801. 10.1158/0008-5472.CAN-15-1326

Mascaux C, Angelova M, Vasaturo A, Beane J, Hijazi K, Anthoine G, Buttard B, Rothe F, Willard-Gallo K, Haller A, Ninane V, Burny A, Sculier JP, Spira A, and Galon J. 2019. Immune evasion before tumour invasion in early lung squamous carcinogenesis. *Nature* 571:570-575. 10.1038/s41586-019-1330-0

Rousseaux S, Debernardi A, Jacquiau B, Vitte AL, Vesin A, Nagy-Mignotte H, Moro-Sibilot D, Brichon PY, Lantuejoul S, Hainaut P, Laffaire J, de Reynies A, Beer DG, Timsit JF, Brambilla C, Brambilla E, and Khochbin S. 2013. Ectopic activation of germline and placental genes identifies aggressive metastasis-prone lung cancers. *Sci Transl Med* 5:186ra166. 10.1126/scitranslmed.3005723

Sanchez-Palencia A, Gomez-Morales M, Gomez-Capilla JA, Pedraza V, Boyero L, Rosell R, and Farez-Vidal ME. 2011. Gene expression profiling reveals novel biomarkers in nonsmall cell lung cancer. *Int J Cancer* 129:355-364. 10.1002/ijc.25704

Wei TY, Hsia JY, Chiu SC, Su LJ, Juan CC, Lee YC, Chen JM, Chou HY, Huang JY, Huang HM, and Yu CT. 2014. Methylosome protein 50 promotes androgen- and estrogen-independent tumorigenesis. *Cell Signal* 26:2940-2950. 10.1016/j.cellsig.2014.09.014

Wei TY, Juan CC, Hisa JY, Su LJ, Lee YC, Chou HY, Chen JM, Wu YC, Chiu SC, Hsu CP, Liu KL, and Yu CT. 2012. Protein arginine methyltransferase 5 is a potential oncoprotein that upregulates G1 cyclins/cyclin-dependent kinases and the phosphoinositide 3-kinase/AKT signaling cascade. *Cancer Sci* 103:1640-1650. 10.1111/j.1349-7006.2012.02367.x

Xu L, Lu C, Huang Y, Zhou J, Wang X, Liu C, Chen J, and Le H. 2018. SPINK1 promotes cell growth and metastasis of lung adenocarcinoma and acts as a novel prognostic biomarker. *BMB Rep* 51:648-653.
